# Flow Cytometry Analysis of Antibacterial Effects of Universal Dentin Bonding Agents on *Streptococcus mutans*

**DOI:** 10.3390/molecules24030532

**Published:** 2019-02-01

**Authors:** Barbara Lapinska, Magdalena Konieczka, Beata Zarzycka, Krzysztof Sokolowski, Janina Grzegorczyk, Monika Lukomska-Szymanska

**Affiliations:** 1 Department of General Dentistry, Medical University of Lodz, 92-213 Lodz, Poland; barbara.lapinska@umed.lodz.pl; 2 Department of Microbiology and Laboratory Medical Immunology, Medical University of Lodz, 92-213 Lodz, Poland; magdalena.konieczka@umed.lodz.pl (M.K.); beata.zarzycka@umed.lodz.pl (B.Z.); janina.grzegorczyk@umed.lodz.pl (J.G.); 3 Department of Conservative Dentistry, Medical University of Lodz, 92-213 Lodz, Poland; krzysztof.sokolowski@umed.lodz.pl

**Keywords:** dental bonding system, flow cytometry, antibacterial activity, *S. mutans*, multi-mode adhesive

## Abstract

There is no consensus on the antibacterial activity of dentin bonding systems (DBS). Many study models have been used to evaluate the antimicrobial activity of dental materials. In this study, a novel detection method, flow cytometry, was introduced. It allows for evaluation of the antibacterial activity of DBS, based on assessment of the disruption of the bacterial physical membrane induced by DBS. The aim of the study was to evaluate the antibacterial properties of selected dentin bonding systems against *Streptococcus mutans*. The highest antibacterial activity against *S. mutans* was observed for Adhese Universal (99.68% dead cells) and was comparable to that of Prime&Bond Universal, OptiBond Universal, or Clearfil Universal Bond Quick (*p* > 0.05). The lowest activity of all tested systems was displayed by the multi-mode adhesive, Universal Bond (12.68% dead bacteria cells), followed by the self-etch adhesive, OptiBond FL (15.58% dead bacteria cells). The present study showed that in the case of two-component DBS, the primer exhibited higher antimicrobial activity than the adhesive (or bond) itself.

## 1. Introduction

Adhesive dentistry relies on the durable interface between (direct or indirect) restorations and hard dental tissues created by means of dental bonding systems (DBS). These systems allow the resin-tooth interface to be sealed by creating a hybrid layer, comprised of a dentinal collagen network infiltrated with adhesive resin.

Commonly used etch-and-rinse adhesives (ERA) present antibacterial activity, mostly due to phosphoric acid etching involved in the adhesive strategy [1,2]. The introduction of self-etch adhesives (SEA) was assumed to overcome the problems with the etch-and-rinse (ER) strategy, i.e., achieving optimal wetness of the dental substrate resulting in inadequate dentin impregnation with adhesive along the resin-dentin interface. Postoperative sensitivity, microleakage, and secondary caries are among the most common clinical implications of the ER technique sensitivity that lead to restorations removal. The mode of action of SEA involves simultaneous demineralization and infiltration of the dentin, resulting in its full infiltration with the adhesive. The self-etch (SE) strategy eliminates etching and rinsing stages. Bacteria left on the cavity surface after preparation may cause damage to the adhesive interface. Some clinicians use various cavity disinfectants, e.g., chlorhexidine (CHX), to remove bacteria prior to the restoration placement. CHX is a widely known disinfectant with proved antibacterial effectiveness against bacteria, including *Streptococcus mutans* [3,4]. Studies have shown that such a procedure might positively influence the dentin surface properties [5] and eventually improve dentin bonding by inhibiting the collagen-bound proteases [6]. However, there is no consensus on it and the synergic effect is product-dependent [7,8,9,10,11,12,13].

*Streptococcus mutans* is one of the crucial pathogens present in the oral cavity [4,14]. This gram-positive bacteria plays a fundamental role in caries development [4] and is present in most carious lesions [15]. It can adhere to hard dental tissues with great potential for biofilm formation due to its high surface energy [16].

The blind spot of the SE strategy was bonding to enamel, which was never as durable as to dentin [17,18]. Therefore, the selective enamel etching (SEE) technique, involving orthophosphoric acid enamel etching and the application of self-etching adhesive on dentin, was introduced [19,20]. The next step in simplifying the dental bonding procedures was the introduction of multi-mode (universal) adhesives (MMA) that could be used in all adhesive strategies: ER, SE, or SEE. It was made possible by the incorporation of functional monomers into the adhesive’s composition [21].

Antibacterial activity of dental bonding systems is in the scope of contemporary adhesive/restorative dentistry. Antibacterial potential of DBS would allow for contamination control on the cavity surface in deep cavities. Such antibacterial activity of DBS depends on several factors, including composition and acidity. Adhesion promoting, acidic monomers, such as N-methacryloyl 5-aminosalicylic acid (5-NMSA) and Phenyl-P—particularly methylene diphosphonate (MDP), containing phosphoric, carboxylic, or acrylic portions (e.g., glutaraldehyde) in the molecules, are considered to be responsible for the antibacterial effect of the primers or adhesive solutions [22]. MDPB monomer is synthesized by combining a methacryloyl group with a quaternary ammonium that gives antibacterial properties and an inhibitory effect against bacterial growth and plaque accumulation [23,24]. DMADDM (dimethylaminododecyl methacrylate), a new quaternary ammonium monomer, inhibited *S. mutans* biofilm growth, lactic acid production, and exopolysaccharides metabolism [25]. Also, it was observed that the acidic nature of self-etching/priming adhesives might be related to their bactericidal effect against *S. mutans* [26].

Microbiological investigations on antibacterial activity of DBS involve an agar diffusion test (ADT) or direct contact test (DCT). The former measures the release of antibacterial substances, while the latter allows the identification of bactericidal or bacteriostatic activity [7,27,28]. Flow cytometry (FC) has been applied in medical diagnostics for many years [29,30,31,32]. Nowadays, flow cytometry as a repeatable, objective, automated diagnostic method is applied in many areas of science and diagnostics, such as in haematology, transplantology, immunology, or microbiology. FC offers wide possibilities for qualitatively and quantitatively analyzing the effects of various agents on microbial cells [33,34,35,36,37]. However, not many dental materials have been investigated using this technique. According to Lukomska-Szymanska et al. [38], flow cytometry application in the evaluation of the antibacterial activity of DBS is a promising diagnostic tool.

The aim of the study was to evaluate the antibacterial properties of selected dentin bonding systems against *S. mutans*.

## 2. Results

**Figure 1:**
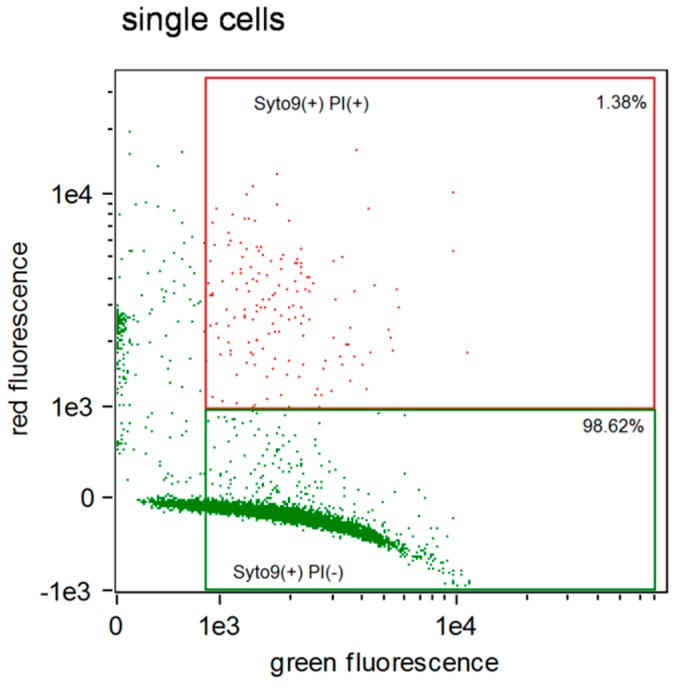
Flow cytometry analysis of *S. mutans* cell-suspension after 1 h incubation with saline (negative control) (single experiment example).

**Figure 2:**
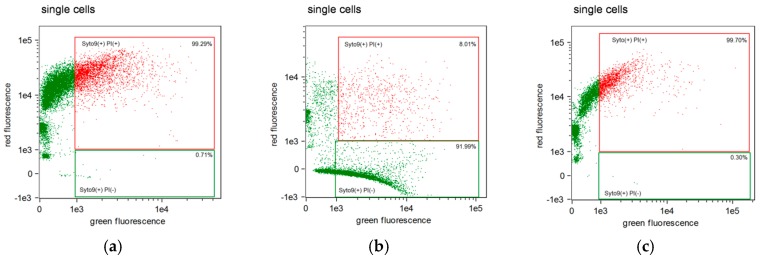
Flow cytometry analysis of *S. mutans* cell-suspension after 1 h incubation with Clearfil SE Bond 2: (**a**) Primer; (**b**) Bond; (**c**) Primer + Bond (single experiment examples).

Representative results of flow cytometry analysis for the antibacterial activity of selected DBS (Clearfil SE Bond 2) on *S. mutans* strain in comparison to a saline control are shown in Figure 1 and Figure 2.

**Figure 3:**
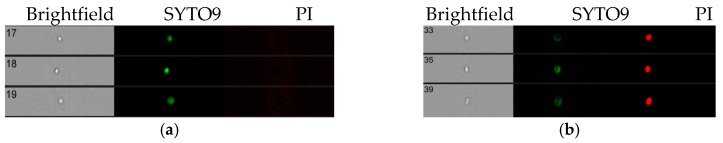
Image gallery of *S. mutans* cells: (**a**) live cells—single stained with SYTO9; (**b**) dead cells–double stained with SYTO9 and PI.

Sample analysis after incubation with saline, as the negative control, shows a very small amount of double stained cells with green and red fluorescence (1.38% dead cells). Almost all cells were single stained with green (SYTO9 + PI−), which means that the majority of cells were alive in this sample (Figure 1 and Figure 3a).

Sample analysis after incubation with Clearfil SE Bond 2 shows differences in antibacterial properties between two utilized components of DBS (Primer and Bond), when these were tested separately. The effect of the Bond component on *S. mutans* cells (Figure 2b) was similar to after incubation with saline (Figure 1), where the majority of cells were alive (8.01% dead cells). However, after incubation with the Primer component, almost all cells of bacteria were double stained (SYTO9 + PI+), which means that cells were dead (99.29% dead cells) (Figure 2a and Figure 3b). The antibacterial activity of Clearfil SE Bond 2 was nearly the same as after *S. mutans* incubation with both components of DBS together in the sample (Prime and Bond). There were 99.70% dead cells (Figure 2c and Figure 3b).

The results shown in Figure 1 and Figure 2 are confirmed by the labelling of cells presented in Figure 3.

Numerical data of dead bacterial cells [%] resulting from the antibacterial activity of DBS are presented in Table 1.

The highest antibacterial activity against *S. mutans* was observed for Adhese Universal (99.68% dead cells) and it was higher than 70% isopropanol activity (positive control). Other universal adhesives, such as Prime&Bond Universal, OptiBond Universal, and Clearfil Universal Bond Quick, also showed very high antibacterial activity against *S. mutans* (96.95–91.13% dead cells, *p* > 0.05). The lowest antibacterial activity was exhibited for Universal Bond (12.68%). When testing the antibacterial activity of single components of a two-component dental bonding system used in the study, OptiBond FL Adhesive (4.65%), followed by Bond of Clearfil SE Bond 2 (5.37%), exhibited the lowest activity against *S. mutans* (Table 1). Table 2 presents the levels of statistical significance for pairwise comparisons of tested adhesives.

## 3. Discussion

The key goal of modern restorative dentistry is minimal invasiveness and the control of bacterial infection of dentin. Various methods of cavity disinfection prior restoration placement have been proposed, i.e., chlorhexidine gluconate (CHX), benzalkonium chlorite-based disinfectant containing ethylenediaminetetraacetic acid (EDTA), and sodium fluoride (NaF). CHX application on a smear layer was introduced prior to bonding with SEAs, as that adhesive strategy had no separate etching and rinsing steps. CHX inhibits a matrix metalloproteinase, preventing hybrid layer degradation and disintegration of the dentin-resin interface [39]. It was reported that the application of 0.2% CHX for 15 s on a dentin surface and draining altered the removal of the smear layer, leaving the surface enriched with CHG deposits [5]. Studies showed that such treatment might adversely affect the dentin bonding ability, with SEAs containing MDP (methacryloyloxi-decyl-dihydrogen-phosphate) [40], the functional monomer responsible for the chemical bond with calcium in HAp [21]. Adhesion-promoting monomers, such as MDP, are usually acidic (hydrogen phosphate or carboxylate) and have a hydrophilic group at one end of the molecule. The high content of such monomers in SEAs lowers the pH values below 3.0 and allows for etching enamel and dentin [27].

DBS and composite restorative materials have gained attention from researchers and manufacturers and an endeavor has been made to incorporate antibacterial components in the composition [7,41,42,43,44,45,46,47,48,49]. The antibacterial effect of adhesives involves disinfecting the cavity and inactivation of any bacteria that might invade the tooth-resin interface via marginal microleakage [27]. Beside antibacterial components such as fluoride, bipyridine, chitosan, chlorhexidine, polyhexanide, amphilic lipids, and silver that might compromise the mechanical properties or bonding effectiveness of resin materials [42,50,51,52,53], antibacterial monomers have been introduced. Among monomers, methacryloxylethyl cetyl dimethyl ammonium chloride (DMAE-CB) and methacyloyloxdodecyl pyridinium bromide (MDPB) present the most prominent antibacterial activity and were incorporated into dental adhesives’ composition [49,54,55,56]. MDPB is an antibacterial monomer (compound of antibacterial agent quaternary ammonium with a methacryloyl group) that is reported to possess significant bactericidal potential against crucial pathogens isolated from dentin and root caries (*S. mutans*, *L. casei*, *L. acidophilus*, and *E. faecalis*) [43,47,48,57,58,59,60,61,62]. The positively charged pyridinium bromide group of MDPB disrupts the negatively charged bacteria cell membrane [18]. Before polymerization, MDPB-containing primer acts as a bactericide solution that disinfects the cavity prepared for restoration [57]. The DMAE-CB monomer contains quaternary ammonium, which exhibits antibacterial activity [63]. The monomer was incorporated in bonding agents (Clearfil Protect Bond), as well as in pit and fissure sealant [64]. Apart from antibacterial monomers, the acidity may influence the antibacterial potential of DBS [8]. Inhibition of bacteria growth has been corresponded with low pH values of the primers [12,60,65,66,67].

Various methods using fluorescent dyes were engaged to evaluate bacterial growth or cell viability, including confocal laser scanning microscopy (CLSM) [58,68,69] and flow cytometry [38]. Additionally, the use of real-time quantitative PCR for glucosyltransferase gene expression in *S. mutans* was reported to evaluate DBS’s antibacterial potential [44,68]. The study used flow cytometry as a well-documented and widely recognized method of assessing the antibacterial properties of various agents. Other commonly used microbiological methods, such as ADT and DCT, deliver inconsistent results on the antibacterial activity of dental adhesives. Vaidyanathan et al. [70] reported that even though some adhesives showed antibacterial activity in the in vitro models (ADT, DCT), the results were not confirmed in the ex vivo model. The researchers argued that the ex vivo model gives more reliable data on the antibacterial activity of dental adhesives than conventional microbiological methods. It was reported that the tooth cavity model, introduced by Ohmori [71] and further modified by Ozer et al. [72], allowed for results comparable to the DCT method to be obtained [73], whereas it may show different results in comparison to the conventional agar well technique [72]. Dentin Disc Technique was introduced as the modification of the agar diffusion method [73]. Furthermore, dentin discs samples may be employed for the evaluation of transdentinal diffusion and cytotoxicity of DBAs [69,74,75,76]. Since a variety of microbiological methods are engaged in the investigation of the antibacterial effects of DBAs’, further studies are required to compare the outcomes of all these methods with flow cytometry.

This study tested Clearfil SE Bond 2, a two-component SEA containing MDP (acidic functional monomer). Studies, using the ADT method, reported that the primer of Clearfil SE Bond (the predecessor of Clearfil SE Bond 2) showed statistically significant antibacterial activity against *S. mutans* [12,77]. However, the adhesive tested alone or a mixture of primer and adhesive exhibited no or very little antibacterial effect [77]. The present study provided contrary findings as Clearfil SE Bond 2 primer, as well as a combination of primer and bond, showed very high antibacterial activity against *S. mutans* (84.02% and 91.78% dead bacteria cells, *p* = 0.121). These results might be explained by the MDP monomer incorporation in both primer and bond composition. The reason behind the small antibacterial effect of the bond itself might be the MDP monomer concentration, which would be lower in the bond than in the primer. Also, HEMA might play a role in the antibacterial effect and overall cytotoxicity of the adhesive. A previous study showed that HEMA was highly released from primer [23]. Literature findings on SEAs’ antibacterial activity against *S. mutans* proved the absence of [10,12,43,78] or very low [8,12,79] antibacterial potential. Research using ADT and DCT methods reported that the bactericidal effect on *S. mutans* only lasted up to 48 h [8,13], indicating possible decomposition of the antibacterial component with time, into surrounding media at different rates [8]. Another study, using both the ADT and the DCT, suggested that the antibacterial effect of the MDPB-containing adhesives (Clearfil Protect Bond) after curing depends on direct contact and does not seem to be related to the diffusion of soluble components [47]. Both the MDPB content and low pH of the primer (pH = 2.0) seemed to be responsible for the antibacterial activity of SEAs [10]. However, it was reported that despite MDPB- and F-content, SEAs did not inhibit caries caused by *S. mutans* [80]. On the contrary, it was found that 300 s after the application of Clearfil SE Bond, as well as 90 s after the application of Clearfil Protect Bond, on dentin, the *S. mutans* viable bacteria count decreased. Since both of these DBSs contain MDP, the antibacterial activity of the monomer, resulting from its low pH (pH = 1.9) was confirmed [58]. Moreover, the antibacterial activity of MDPB-containing SEAs lasted for approximately one week [81]. OptiBond FL, 3-step ERA, was recognized as the gold standard of its class as it showed high microtensile bond strength, low nanoleakage, and a high degree of conversion [82]. It was proven to produce a stable bond to hard dental tissues due to a peripheral enamel acid-etched resin seal [83]. According to the manufacturer’s instructions, OptiBond FL releases fluoride. Previous studies using the ADT method showed that OptiBond FL exhibited no antibacterial activity against *S. mutans*, which could be attributed to the lack of a monomer and high pH value [84]. OptiBond FL does not contain MDP, but HEMA and glycerol in primer. Findings of the present study using flow cytometry confirmed the low antibacterial activity of OptiBond FL (15.58% dead bacteria cells). However, when the two components of the DBS were tested independently, it was found that the primer exhibited high antibacterial activity (94.18% dead bacteria cells), whereas the adhesive lacked such activity (4.65% dead bacteria cells). The differences in antibacterial properties could result from the differences in the pH value of the primer and adhesive, with values of 1.9(2.0) and 6.9(5.0), respectively. Other studies using ADT confirmed the higher antibacterial effect against *S. mutans* of the primer than of the adhesive of OptiBond FL, reporting that the mixture (primer & adhesive) showed no antibacterial effect at all [85]. The authors suggested that since, after polymerization, OptiBond FL showed no antibacterial activity, the antibacterial effect of the unpolymerized bonding system might be attributed to HEMA elution and its cytotoxic potential [85].

A previous study, using flow cytometry, showed that, in general, SEAs exhibited significantly higher antimicrobial activity against *E. faecalis* than ERAs [38]. In the present study, using flow cytometry, corresponding results were obtained. Antibacterial activity against *S. mutans* of SEA, Clearfil SE Bond 2, was significantly higher than of ERA, OptiBond FL (91.78% vs. 15.58% dead bacteria cells). Both DBS are two-component adhesives. The comparison of antibacterial activity of the primers showed no statistically significant difference (*p* = 0.270), though it was a little higher for the OptBond FL primer (94.18% vs. 84.02% dead bacteria cells). Contrary findings were presented by Başeren et al. [86], who, based on the ADT method, reported that the Clearfil SE Bond primer had a stronger effect on *S. mutans* than the OptiBond FL primer, despite having comparable pH values (pH = 2.0). The authors argued that it might be the MDP incorporation in Clearfil SE Bond that influenced the reduction of bacteria counts. The present study using flow cytometry did not confirm the findings obtained by the ADT method.

The clinical performance of the SE strategy was enhanced with additional etching of enamel (SEE). Recently, MMAs gained popularity due to their versatile indications for use, ranging from enamel and dentin bonding using the ER, SE, and SEE strategy up to bonding to glass ceramics and zirconia [87,88]. They comprise two different approaches to creating an adhesive layer [19]. Universal adhesives’ matrix is based on a combination of monomers of hydrophilic (hydroxyethul methacrylate/HEMA), hydrophobic (decandiol dimethacrylite/D3MA), and intermediate (bis-GMA) nature, allowing for bonding between the hydrophilic tooth substrate and hydrophobic resin restorative, under a variety of surface conditions [89].

The present study investigated several MMAs (Adhese Universal, Prime&Bond Universal, OptiBond Universal, and Clearfil Universal Bond Quick); for most of them, the highest antibacterial effect against *S. mutans* was observed. These high values might be attributed to the MMAs’ composition. Incorporation of acidic primers (the phosphonic acid functional groups) in Adhese Universal and Prime&Bond Universal composition (methacrylated phosphoric acid ester and phosphoric acid modified acrylate resin, respectively) (Table 3) raised the adhesives’ acidity [90]. A previous study using flow cytometry showed that ERA containing phosphoric acid modified acrylate resin (Prime&Bond one Etch&Rinse) exhibited moderate antibacterial activity against *E. faecalis* (72.04% dead bacteria cells) [38]. OptiBond Universal, likewise the OptiBond FL Primer, showed a high antibacterial effect on *S. mutans*, which may be attributed to the HEMA and glycerol content. Monoalcylglycerols were reported to inhibit *S. mutans* biofilm formation [91], whereas cytotoxic HEMA possibly diffused from polymerized MMA, producing a bactericidal effect. Clearfil Universal Bond Quick is a fluoride releasing, MDP-containing MMA with a pH value of 2.3. Its high antibacterial activity against *S. mutans* (91.13% dead bacteria cells) observed in the present experiment could be attributed to both abovementioned components. The lowest antibacterial activity among tested MMAs was observed for Universal Bond (12.68% dead bacteria cells). The result might be explained by the low concentration of acidic monomers in the composition.

Almost all tested MMAs showed great antibacterial potential against *S. mutans*, which might result from the incorporation of acidic monomers in their composition (and low pH value); fluoride release; or, in general, the cytotoxicity of the components. It was reported that universal adhesives should not be recommended for use in deep cavities due to their high cytotoxic potential to pulp cells, regardless of the application (etching) mode [75]. SEAs (Clearfil SE Bond and Xeno V), as well as ERA (Adper Single Bond Plus), were shown to reduce cells viability (determined by enzyme activity) by 50% [76]. The transdentinal diffusion of monomers such as HEMA, bis-GMA, or camphorquinone [74,85,92], as well as UDMA and TEGDMA [93], the main components of tested adhesives, is responsible for DBSs’ high cytotoxicity. Some researchers proposed replacing the HEMA in SEAs with surfactant di-methacrylate monomers [94]. For most tested experimental, HEMA-free adhesives (containing monomers Bis-EMA 10, Bis-EMA 30, PEG 400, PEG 400 UDMA, or PEG 1000), a higher cell viability was observed in comparison to the HEMA-containing adhesive. Also, no relevant difference in the degree of conversion was reported, creating a promising alternative for adhesives’ formulations yielding a less cytotoxic effect on pulp cells.

The literature presents inhomogeneous and limited findings on the antibacterial properties of DBS, and MMAs in particular, as they are a relatively new group of bonding systems [64,89,95]. The clinical performance of MMAs applied in non-carious cervical lesions was found to be promising in a six-month evaluation, irrespective of the employed bonding strategy [96,97]. However, it was shown, that using MMAs in SE mode, in both non- and carious lesions, led to producing marginal discoloration or degradation of marginal adaptation in 18- to 24-month clinical follow-up [98,99,100]. In the case of bonding to the enamel/dentin of permanent teeth, the SEE strategy was recommended, yet long-term clinical studies are lacking [101]. Further studies should be carried out to assess the antibacterial effect of MMAs’ components and their potential diffusion from polymerized universal dental adhesives.

## 4. Materials and Methods 

### 4.1. Eluate Preparation

The dental bonding systems used in the study are presented in the Table 3.

All DBS were added in a volume of 50 µL to separate U-bottom shaped tubes (16 mm in diameter) and distributed evenly. Then, DBS were polymerized according to the manufacturer’s instructions and 2 mL sterile buffered saline (OXOID, GB) was aliquoted and incubated for 24 h in 35 °C. The day after, each sample was centrifuged (2000 rpm, 5 min) to obtain eluates utilized in further experiments.

### 4.2. Microbank System

Microbiological studies were conducted on the reference strain *Streptococcus mutans* ATCC 25175. The strain was stored in the Microbank system (Biocorp, Warszawa, Poland) in cryopreservation medium in a freezer at −80°C, as described by Lukomska-Szymanska et al. [42].

### 4.3. Bacteria Suspension Preparation

The *S. mutans* strain was revived from the Microbank system on proliferating medium, Columbia agar (Becton Dickinson, Franklin Lakes, NJ, USA), under aerobic conditions in 35 °C. After the first 18-h cultivation, the next 18-h bacterial culture was produced at the new medium plate to obtain reproducibility of the method. Each experiment was performed from the same, second recultivation. The bacterial colonies harvested from the medium were used to gain suspension in McFarland standard 0.5 in sterile buffered saline. The effect of each DBS (Table 3) was assessed on a bacteria suspension.

### 4.4. Bacteria Incubation

A 1 mL aliquot of the bacterial suspension was added into 13 sterile tubes and centrifuged at 10,000 g for 2 min. The supernatants were discarded and replaced respectively with 1 mL: 0.85% NaCl (negative control), 70% isopropanol (positive control), or the eluate (test samples) prepared by the bonding system. After resuspension of the pellets to homogeneous suspension, samples were incubated for 1 h in 35 °C and well-mixed every 15 min. Next, all samples were centrifuged (10,000 g, 2 min) and washed with phosphate buffered saline (PBS) without Ca and Mg ions (PAN Biotech, Aidenbach, Germany). After the washing step, 300 µL aliquot of PBS was added to the pellet of bacteria. Each sample was analyzed with the LIVE/DEAD flow cytometry method.

### 4.5. Fluorescent Staining Procedure

The method was described in detail by Lukomska-Szymanska et al. [38]. The LIVE/DEAD^®^ BacLight^TM^ Bacterial Viability Kit (Molecular Probes, Life Technologies, Eugene, OR, USA) was used for the analysis, according to the manufacturer’s instructions. A total of 150 µL of the bacteria suspension from each sample was stained with 5 µL SYTO9 and propidium iodide (PI) and incubated for 15 min in the dark at room temperature. Flow cytometric measurements were performed on ImageStreamX Mark II (ISX-MkII) (Amnis, EMD Millipore, Seattle, WA, USA) with 488 nm excitation from a blue laser, at 50 mW, counting 10,000 objects. The fluorescence signals were displayed in the green and red channels. The microbial cells with an intact cell membrane show green fluorescence (live cells), whereas the bacteria with damaged cytoplasmic membranes exhibit green and red fluorescence at the same time (dead cells). Results were expressed as the percentage of dead bacterial cells and were analyzed using IDEAS^®^ 6.1 (Image Data Exploration and Analysis Software). Each experiment was performed in six replications. For the single-colour histogram charts, representative experiments are shown in Figure 1 and Figure 2. The numeric results including standard deviation are listed as a mean (Table 1).

### 4.6. Statistical Analysis

The numerical data, i.e., percentage of dead bacteria cells, were collected and statistically analyzed. The dental bonding systems used in the study were codified as a discrete variable. Due to the small sample sizes, and eventually an abnormal distribution of the numerical data, non-parametric tests were performed. The Kruskal-Wallis rank test was performed to test the significance of differences between the investigated bonding systems. In order to enhance the statistical power of the computations and diminish a faulty inference, the exact variant of the Kruskal-Wallis rank test was harnessed. Afterwards, post hoc pairwise comparisons, based on Fisher’s protected least-significant difference (LSD), were performed in order to identify statistically meaningful differences in all pairs of measurements. A level of *p* < 0.05 was considered statistically significant. All the statistical procedures were carried out using Stata^®^ (Stata/Special Edition for Windows, release 14.2, Publisher: StataCorp LP, College Station, TX, USA, 2018).

## 5. Conclusions

The present study showed the great antibacterial potential of universal bonding systems against *S. mutans*. Such activity might be attributed to incorporation of the MDP monomer in DBSs’ composition or, in general, to the cytotoxicity of the components. Further studies should be carried out to investigate which components are responsible for the antibacterial effect of DBSs’ not containing the MDP monomer.

## Figures and Tables

**Table 1:** Antibacterial activity of DBS against *S. mutans*—statistical parameters (M = mean values [%], SD = standard deviation, CV = coefficient of variation).

	DBS	M	SD	CV	Min.–Max.
1.	Adhese Universal	99.68	0.25	0.25%	99.19–99.87
2.	Clearfil Universal Bond Quick	91.13	12.91	14.17%	65.62–99.94
3.	Clearfil SE Bond 2 (Primer)	84.02	15.96	18.99%	56.87–99.33
4.	Clearfil SE Bond 2 (Bond)	5.37	4.33	80.70%	1.96–13.04
5.	Clearfil SE Bond 2 (Primer + Bond)	91.78	12.10	13.18%	68.57–99.82
6.	OptiBond Universal	94.99	7.87	8.28%	79.55–99.84
7.	OptiBond FL (Primer)	94.18	7.32	7.77%	80.00–99.87
8.	OptiBond FL (Adhesive)	4.65	3.23	69.47%	1.85–10.66
9.	OptiBond FL (Primer + Adhesive)	15.58	14.01	89.95%	5.95–42.96
10.	Prime&Bond Universal	96.95	4.11	4.24%	88.64–99.39
11.	Universal Bond	12.68	12.40	97.77%	3.37–34.32
12.	Isopropanol 70%	97.58	3.40	3.49%	88.95–99.82
13.	NaCl 0.85%	2.73	2.25	82.55%	1.16–8.96

**Table 2:** Levels of statistical significance for post hoc pairwise comparisons of dentin bonding systems (DBS), based on Fisher’s protected least significant difference (LSD).

DBS No.	1.	2.	3.	4.	5.	6.	7.	8.	9.	10.	11.	12.	**13.**
**1.**													
**2.**	*p* = 0.088												
**3.**	***p* = 0.002**	*p* = 0.154											
**4.**	***p* < 0.001**	***p* < 0.001**	***p* < 0.001**										
**5.**	*p* = 0.114	*p* = 0.897	*p* = 0.121	***p* < 0.001**									
**6.**	*p* = 0.347	*p* = 0.437	***p* = 0.029**	***p* < 0.001**	*p* = 0.517								
**7.**	*p* = 0.270	*p* = 0.539	***p* = 0.043**	***p* < 0.001**	*p* = 0.627	*p* = 0.870							
**8.**	***p* < 0.001**	***p* < 0.001**	***p* < 0.001**	***p* = 0.042**	***p* < 0.001**	***p* < 0.001**	***p* < 0.001**						
**9.**	***p* < 0.001**	***p* < 0.001**	***p* < 0.001**	***p* = 0.042**	***p* < 0.001**	***p* < 0.001**	***p* < 0.001**	***p* = 0.030**					
**10.**	*p* = 0.583	*p* = 0.243	***p* = 0.011**	***p* < 0.001**	*p* = 0.298	*p* = 0.693	*p* = 0.577	***p* < 0.001**	***p* < 0.001**				
**11.**	***p* < 0.001**	***p* < 0.001**	***p* < 0.001**	*p* = 0.143	***p* < 0.001**	***p* < 0.001**	***p* < 0.001**	*p* = 0.108	*p* = 0.560	***p* < 0.001**			
**12.**	*p* = 0.631	*p* = 0.142	***p* = 0.003**	***p* < 0.001**	*p* = 0.186	*p* = 0.554	*p* = 0.437	***p* < 0.001**	***p* < 0.001**	*p* = 0.886	***p* < 0.001**		
**13.**	***p* < 0.001**	***p* < 0.001**	***p* < 0.001**	*p* = 0.539	***p* < 0.001**	***p* < 0.001**	***p* < 0.001**	*p* = 0.653	***p* = 0.004**	***p* < 0.001**	***p* = 0.023**	***p* < 0.001**	

The bold indicates the statistically significant *p* values.

**Table 3:** Dental bonding systems used in the study.

Name	Manufacturer	Number of Components	Type	Composition	Mode of Etching		
					ER ^1^	SE ^2^	SEE ^3^
Adhese Universal	Ivoclar Vivadent/Germany	1	1-step	methacrylated phosphoric acid ester (MDP, 3–10%), MCAP methacrylated carboxylic acid polymer, HEMA (10–25%), Bis-GMA (10–25%), D3MA (Decandiol dimethacrylate) (3–10%), 2-dimethylaminoethyl methacrylate (1–2.5%), camphoroquinone (1–2.5%), ethanol (10–25%)	+	+	+
Clearfil Universal Bond Quick	Kuraray America/USA	1	1-step	10-MDP, Bis-GMA (10–25%), HEMA (2.5–10%), ethanol (10–25%) Hydrophilic amide monomers, Colloidal silica, Silane coupling agent, Sodium fluoride, dl-Camphorquinone	+	+	+
Clearfil SE Bond 2 (Primer+Bond)	Kuraray America/USA	2	2-step	Primer: 10-MDP, HEMA (20–40%), Hydrophilic aliphatic dimethacrylate, dl-Camphorquinone Bond: 10-MDP, Bis-GMA (25–45%), HEMA (20–40%), Hydrophobic aliphatic dimethacrylate, dl-Camphorquinone, Silanated colloidal silica		+	
OptiBond Universal	Kerr/USA	1	1-step	Acetone (30–60%), HEMA (5–10%), Glycerol Dimethacrylate (1–5%), ethanol (5–10%)	+	+	+
OptiBond FL (Primer+Adhesive)	Kerr/USA	2	3-step	Primer: HEMA (10–30%), ethanol (10–30%), 4-MET (10–30%), glycerol phosphate dimethacrylate (5–10%) Adhesive: HEMA (10–30%), ytterbium trifluoride, 3-trimethoxysilylpropyl methacrylate (5–10%), 2-hydroxy-1,3-propanediyl bismethacrylate (5–10%), alkali fluorosilicates(Na) (1–5%)	+		
Prime&Bond Universal	Dentsply/UK	1	1-step	Phosphoric acid modified acrylate resin, Multifunctional acrylate, Bifunctional acrylate, Acidic acrylate, Isopropanol, Water, Initiator	+	+	+
Universal Bond	Tokuyama/Japan	2	1-step	Phosphoric acid monomer (1–5%), Bisphenol A, Bis-GMA, TEGDMA, HEMA (10–30%), MTU-6 (thiouracil monomer), Silane coupling agent, Peroxide, Borate catalyst, Acetone, Isopropanol	+	+	+

^1^ ER = etch-and-rinse; ^2^ SE = self-etch; ^3^ SEE = selective enamel etching.
